# Exploring Factors Associated with Prescribers’ Comfort Levels in Analgesic Prescribing in Quebec

**DOI:** 10.2147/JPR.S469052

**Published:** 2024-08-16

**Authors:** Usra Naeem, Gwenaelle De Clifford-Faugère, Marimée Godbout-Parent, Hermine Lore Nguena Nguefack, Anaïs Lacasse

**Affiliations:** 1 Département des sciences de la santé, Université du Québec en Abitibi-Témiscamingue, Rouyn-Noranda, Quebec, Canada; 2 Department of Health Professional Technologies, Faculty of Allied Health Sciences, University of Lahore, Lahore, Pakistan

**Keywords:** medications, pharmacological treatment, healthcare professionals, physician, pharmacist, nurse, factors, sex

## Abstract

**Purpose:**

Identifying the factors associated with comfort level when prescribing medications is important for tailoring education and training. This study aimed to explore factors associated with the comfort level of healthcare professionals regarding dispensing and adjusting prescriptions for the treatment of chronic pain (CP).

**Methods:**

A cross-sectional survey was conducted among licensed physicians, pharmacists, and nurse practitioners across the province of Quebec, Canada. Comfort level regarding dispensing and/or adjusting prescriptions for CP treatment was measured on a 0–10 rating scale (0 = very uncomfortable, 10 = very comfortable).

**Results:**

In total, 207 prescribers participated in this study (83 physicians, 58 pharmacists, and 66 nurse practitioners). 56.5% reported a comfort level in dispensing and/or adjusting prescriptions for the treatment of CP <6/10. The median comfort level score was 6 (interquartile range - IQR: 2). Differences in median scores were found between physicians (6), pharmacists (7) and nurses (5; p < 0.001). Multivariable logistic regression revealed that the factors associated with an increased likelihood of reporting a high comfort level (≥6/10) were: being a pharmacist, having a relative living with CP, a greater percentage of past year continuing educational activities about CP management, and higher perception of short-acting opioids risks. Factors associated with lower comfort levels were as follows: being a nurse practitioner, having fewer years of experience, living in a remote region, living with CP, and a higher perception of long-acting opioids risks. The practice setting and sex at birth were also associated with comfort level.

**Conclusion:**

The comfort level regarding prescribing for CP varies according to socioeconomic/professional factors, which can lead to disparities in the quality of care and outcomes for patients. Our results reinforce the importance of investing in initial training and continuing education of prescribers.

## Introduction

Chronic pain (CP) affects more than 20% of the population and nearly 8 million people in Canada are living with CP, 1,2 which is characterized as the pain that persists or recurs for more than 3 months.^3^ CP is one of the most common reasons why people seek medical care.^4^ It exerts an enormous financial burden not only on people living with this condition but also on the healthcare system of a country.^1^ People living with CP report significant impact of this disease on their physical functioning, social relationships, mental health, and overall quality of life.^1^,^5^,^6^

CP is a complex and multidimensional phenomenon.^7^ The cause of CP often lacks objective findings such as direct tissue injury.^8^ The biopsychosocial model of pain defines CP as the interplay of biological, psychological, and social factors.^7^ Optimal management of CP requires an interdisciplinary approach that includes physicians, pharmacists, nurses, physiotherapists and other allied healthcare professionals.^1^ It is noteworthy that not only patient-related factors are associated with pain perception and its treatment,^9^ but also factors associated with healthcare professionals, such as their sex at birth, experience, medical training and work environment.^10^,^11^ Management of this condition is challenging due to certain factors: poor recognition of this medical condition, underutilization of pain assessment tools, limited resources for the field of pain medicine (particularly outside large urban areas), lack of clinical guidelines for the management of CP or lack of awareness of these clinical guidelines among healthcare professionals, inadequate training of healthcare professionals, and concerns regarding adverse effects of medications used for the management of CP such as opioids.^8^,^12–14^

Owing to the widespread occurrence of this medical condition, it is warranted that healthcare professionals possess a high level of confidence and expertise in treating patients with CP, potentially leading to better quality of life among patients. However, many studies have reported a lack of confidence and dissatisfaction among healthcare professionals when it comes to treating patients with CP.^15–17^ There is a need to enhance the clinical competencies of healthcare professionals when dealing with individuals experiencing CP along with identifying factors that could boost their confidence while managing these patients. Studying the comfort level of healthcare professionals regarding prescribing medications for CP and associated factors is crucial for patient safety, optimal treatment outcomes, shared decision-making, risk mitigation, and continuous improvement in clinical practices.^15^,^18^ Also, it provides valuable insights that can inform targeted interventions and enhance healthcare professionals’ confidence in providing effective and safe pain management for their patients.^15^ In addition, identifying factors associated with the comfort level of healthcare professionals in prescribing medication for CP is equally important for understanding barriers, tailoring education and training. By addressing these factors, healthcare systems can potentially enhance the comfort level of healthcare professionals, optimize pain management practices, and ultimately improve patient outcomes.

The objective of this study was to explore the sociodemographic and professional characteristics associated with the comfort level of healthcare professionals regarding dispensing and adjusting prescriptions for the treatment of CP across the province of Quebec, Canada. Physicians, pharmacists, and nurse practitioners were specifically targeted, as they were the three healthcare professionals authorized to prescribe pain medications in Canada.

## Methods

### Prescribing Context

In Canada and Quebec, all medication classes that can be used for CP management (eg, anti-inflammatory drugs, opioids, antidepressants, anticonvulsants, muscle relaxants) can be prescribed by physicians, but also by nurse practitioners (initiated and adjusted).^19^ In Quebec, pharmacists can adjust prescriptions, including pain medications.^20^ As of now, pharmacists cannot initiate a medication for pain but can modify (adjust the dosage, form, or dose; extend an existing prescription) or discontinue the drug therapy to ensure patient safety or effectiveness. This includes designated substances (narcotics). However, in this context, the modification cannot exceed the originally prescribed total quantity.^20^ Medical cannabis can be authorized by physicians, and in some provinces such as Quebec, by nurse practitioners.^19^,^21^

### Study Design and Data Collection

This cross-sectional study stems from the secondary analysis of the database of a project focusing on clinicians’ perceptions regarding the risk associated with different medications used by persons living with CP.^22^ An online questionnaire was used to collect data from 1st March until 28th May, 2022. The inclusion criteria for the participants were as follows: 1) being a physician, pharmacist, or nurse practitioner dispensing and/or adjusting prescriptions for the treatment of CP in their clinical practice; 2) having a valid license to practice in Canada; and 3) being able to complete an online questionnaire in French. The recruitment strategy used various online platforms, including social media, webpages, and e-newsletters of different Quebec professional associations and research networks. Invitations to participate in the study were also disseminated via Email by members of the research team. The invitation across all dissemination platforms included a URL that directed potential participants to an anonymous online questionnaire hosted on a SurveyMonkey^®^ platform. The landing page of the questionnaire ensured that participants could provide informed consent electronically. The complete methodology is described in detail elsewhere.^22^

### Measured Variables

The dependent variable for the present study was the comfort level of prescribers in dispensing and/or adjusting prescriptions for the treatment of CP, measured on a numerical rating scale (NRS) from 0 to 10 (0 = very uncomfortable and 10 = very comfortable). Studying overall healthcare professionals’ comfort level in prescribing analgesics was considered relevant in the real-world context of chronic pain management where pain pharmacotherapy is often multimodal (are they adequately prepared to support patients?). Independent variables included the perception of respondents’ risk in prescribing short-acting opioids and long-acting opioids for the treatment of CP, which was also measured using two 0–10 NRS (0 = no risk, 10 = very high risk). The definition of the overall risk of medication was specified to participants before survey completion, that is, overall risk of medication to cause a short- or long-term adverse reactions, such as organ-specific or systemic toxicity (gastrointestinal symptoms, central nervous system), medication interactions, physical/psychological dependence potential, abuse potential, insomnia, tolerance, increased pain perception over time (hyperalgesia), and memory or concentration problems. Such risk perceptions were included as they were expected to significantly impact the overall comfort level in dispensing and adjusting prescriptions for CP. Given the expected variability, both long-acting and short-acting opioids were considered. Sociodemographic and other characteristics of the prescribers analyzed were as follows: sex at birth, gender identity, years of clinical experience, type of practice (eg, primary care clinic, hospital, emergency room, community pharmacy, pain clinics), and region of practice (the 17 administrative regions of Quebec were categorized as remote regions and regions near large urban centers). In addition to these items, two closed-ended questions were also asked to participants: if they lived with CP (pain for more than 3 months), and if any relatives of the respondent lived with CP. We also collected data on the percentage of continuing educational activities that were about the management of CP in the past year (measured on NRS scale from 0% to 100%).

### Statistical Analysis

We used the frequency distribution (n, %) to describe the sociodemographic characteristics of the respondents. The comfort level of prescribers in dispensing and/or adjusting prescriptions for the treatment of CP (0–10) is described as medians, interquartile ranges (IQR), means, and standard deviations (SD). First, comfort level scores were compared between the groups formed by our independent variables using bivariable analyses. Normal distribution of data was tested using Shapiro–Wilk normality tests, and Mann–Whitney U and Kruskal–Wallis tests were applied, given that the normality assumption was not satisfied. For continuous independent variables (short-acting opioids risk perception, long-acting opioids risk perception, and percentage of continuing educational activities about the management of CP), the correlation with comfort level scores was assessed using Spearman’s rank correlation coefficients (rho). Statistical significance was set at p < 0.05. We then used multivariable logistic regression to identify the characteristics of respondents that were associated with their comfort level in dispensing and/or adjusting prescriptions for CP treatment. For the logistic regression analysis, comfort level was coded as a dichotomous outcome variable, that is, comfort level <6 and ≥6. This cutoff value of 6 was selected as it represents the median comfort level score in our sample. The assumption of linearity of the independent variables and log-odds were assessed using the Box–Tidwell method. Multicollinearity was assessed using the variance inflation factor (VIF). Sex at birth was chosen instead of gender identity for the multivariable model. The Hosmer–Lemeshow test was used to assess the goodness-of-fit of the model. Results are presented in the form of odds ratios and 95% confidence intervals. All the above-mentioned variables were a priori selected through literature review and clinical considerations. Multiple imputation method was used to handle missing data. Sensitivity analysis was achieved to assess if our conclusions remained consistent when the comfort level was analyzed as a continuous variable using linear regression. All statistical analyses were conducted using IBM SPSS Statistics 28^®^ (IBM Corp: Armonk, NY). No a priori sample size calculation was achieved as we conducted a secondary analysis of a previous dataset (convenience sample).

## Results

The detailed characteristics of the participants are presented in Table 1. A total of 207 individuals completed the questionnaire, of which 83 were physicians, 58 were pharmacists, and 66 were nurse practitioners. A majority of respondents were female in terms of sex at birth (86.0%), and gender identity differed from sex at birth for 0.5% of our sample (one person reported not identifying with any of the options offered in the questionnaire). Fifty-seven percent of respondents had more than 11 years of experience in pain management, and 44.0% were practising in primary care clinics. The majority were practising in non-remote regions (84.5%) of the province. Most healthcare professionals (56.5%) reported a comfort level in dispensing and/or adjusting prescriptions for the treatment of CP <6/10.

**Table 1:** Sociodemographic Characteristics of Participants (n = 207)

Variables	Frequency^a^	Proportion (%)
**Sex at birth**		
Female	178	86.0
Male	29	14.0
**Gender Identity**		
Women	177	85.5
Men	29	14.0
Other^b^	1	0.5
**Type of Profession**		
Physician	83	40.1
Nurse Practitioner	66	31.9
Pharmacist	58	28.0
**Years of Experience**		
0–5	52	25.1
6–10	36	17.4
11+ years	119	57.5
**Type of Practice**		
Primary care clinic	91	44.0
Hospital or Emergency room	47	22.7
Community pharmacy	41	19.8
Pain Clinic	9	4.3
Other^c^	19	9.2
**Region**		
Remote^d^	32	15.5
Non-remote	175	84.5
**Living with chronic pain**		
Yes	57	27.5
No	150	72.5
**Relatives living with chronic pain**		
Yes	77	62.8
No	130	37.2

**Notes**: ^a^ 1.4 to 4.8% missing data across independent variables. ^b^ One participant reported not identifying with any of the options mentioned in the survey. The options offered were man, woman, non-binary, trans-man, trans-woman, and two-spirited. ^c^ Other settings included local community service centers, long-term care residences, outpatient clinics, rehabilitation centers, operating rooms, palliative care, and telemedicine. ^d^ Remote resource regions as defined by Revenu Quebec (ie, the provincial revenue agency): Bas-Saint-Laurent, Saguenay-Lac-Saint-Jean, Abitibi-Témiscamingue, Côte-Nord, Nord-du-Québec, and Gaspésie-Îles-de-la-Madeleine. Non-remote regions are located near major urban centers.

Summary statistics of the comparison of the comfort level of healthcare professionals across subgroups based on independent variables is presented in Table 2. The median comfort level score of healthcare professionals in dispensing and/or adjusting prescriptions for CP treatment was 6 (IQR: 2). Bivariable analyses revealed statistically significant differences in comfort level between subgroups formed according to sex at birth, years of experience, and type of healthcare professional; males, more experienced clinicians, and pharmacists being more comfortable in dispensing and/or adjusting prescriptions for the treatment of CP (Table 2). There was a positive correlation between the comfort level of respondents and the percentage of time spent on continuing educational activities about the management of CP (Spearman rho = 0.465, p < 0.001). The median score for the perception of risk associated with long-acting and short-acting opioids was 8, and we did not find any correlation between the comfort level of healthcare professionals and the perception of risk associated with long-acting and short-acting opioids in the bivariable analysis. If Bonferroni had been applied, the adjusted significance level for interpretation would have been 0.005 (0.05 divided by 10 tests), which would not alter the conclusions drawn from our bivariable tests.

**Table 2:** Comparison of Difference in Comfort Level of Healthcare Professionals in Dispensing and/or Adjusting Prescriptions for the Treatment of Chronic Pain (Bivariable Comparisons; n = 207)

Variables	Comfort Level Mean ± S. D	P-value
**Sex at birth**		
Female	5.8±1.90	**<0.001**
Male	7.14±1.45	
**Type of Profession**		
Physician	6.43±1.82 ^a^	**<0.001**
Nurse Practitioner	5.11±1.75 ^a^	
Pharmacist	6.59±1.77 ^a^	
**Years of Experience**		
<11 years	5.48±1.77	**<0.001**
≥11 years	6.48±1.87	
**Type of Practice**		
Primary care clinic	5.90±1.70	0.215
Hospital or Emergency room	5.83±2.27	
Community pharmacy	6.29±1.80	
Pain Clinic and other*^b^*	6.57±1.85	
**Region**		
Remote^c^	5.81±1.63	0.483
Non-remote	6.10±1.94	
**Living with chronic pain (Yes/No)**		
Yes	6.07±1.94	0.983
No	6.05±1.78	
**Relatives living with chronic pain (Yes/No)**		
Yes	5.91±1.80	0.423
No	6.17±1.94	

**Notes**: The level of significance was set at ˂0.05. Statistically significant p values are shown in bold. SD, Standard deviation. ^a^ As the present study stems from a secondary analysis of a previous project, the mean comfort level scores (and standard deviations) were previously published to describe the study population.^23^ bOther settings included local community service centers, long-term care residences, outpatient clinics, rehabilitation centers, operating rooms, palliative care, and telemedicine. ^c^ Remote resource regions as defined by Revenu Quebec (ie, the provincial revenue agency): Bas-Saint-Laurent, Saguenay-Lac-Saint-Jean, Abitibi-Témiscamingue, Côte-Nord, Nord-du-Québec, and Gaspésie-Îles-de-la-Madeleine. Non-remote regions are located near major urban centers.

The results of the multivariable logistic regression analysis are summarized in Table 3. The results of the variance inflation factor (VIF) showed that multicollinearity was unlikely to exist among the variables included in the final model (VIF between 1.067 and 3.59). The Hosmer–Lemeshow test supported the goodness-of-fit of the model (p > 0.05). The factors that were associated with an increased likelihood of reporting higher comfort level in dispensing and/or adjusting prescriptions for the treatment of CP were: 1) being pharmacist (adjusted OR pharmacists vs physicians: 3.98, 95% CI: 2.2–7.13), 2) practising at a pain clinic (adjusted OR pain clinic vs primary care clinic: 1.17, 95% CI: 1.10–2.73), 3) having a relative living with CP (adjusted OR: 1.41, 95% CI: 1.04–1.09), 4) the percentage of time spent on continuing educational activities about the management of CP in the past year (adjusted OR: 1.06, 95% CI: 1.04–1.06), and 5) higher perception of short-acting opioids risks (adjusted OR: 1.15, 95% CI: 1.00–1.31). Factors associated with lower comfort levels were: 1) being a nurse practitioner (adjusted OR nurses vs physicians: 0.27, 95% CI: 0.18–0.38), 2) less than 11 years of clinical experience (adjusted OR: 0.31, 95% CI: 0.23–0.43), 3) practising in remote regions (adjusted OR: 0.78, 95% CI: 0.52–1.15), 4) living with CP (adjusted OR: 0.70, 95% CI: 0.51–0.97). Practice setting was also associated with comfort level, but not sex at birth or gender identity. Appendix 1 shows results for original data and data with imputed values. In the sensitivity analysis, the use of a linear regression still revealed statistically significant associations between comfort level and perception of opioids risks, continuing educational activities about the management of CP, years of clinical experience, type of healthcare professional, and type of practice. However, it allowed capturing an association which was not captured in the logistic multivariable model, ie, with sex at birth.

**Table 3:** Associations Between Respondent Characteristics and a Reported Comfort Level in Dispensing and/or Adjusting Prescriptions for the Treatment of Chronic Pain ≥6/10 (Multivariable Logistic Regression Model; n = 207)

Variables	Adjusted OR (95% CI)	P-value
Sex (male vs female)	1.46 (0.98–2.19)	0.06
Type of profession (vs Physicians)		
Nurse practitioners	0.27 (0.18–0.38)	**<0.05**
Pharmacists	3.98 (2.2–7.13)	**<0.05**
Years of experience (0–10 years vs 11+ years)	0.31 (0.23–0.43)	**<0.05**
Type of practice (vs primary care clinic)		
Hospital & emergency room	0.37 (0.24–0.51)	**<0.05**
Community pharmacy	0.46 (0.24–0.87)	**<0.05**
Pain clinic and other^a^	1.17 (1.10–2.73)	**<0.05**
Region (remote^b^ vs non-remote)	0.78 (0.52–1.15)	0.213
Living with chronic pain	0.70 (0.51–0.97)	**<0.05**
Having a relative living with chronic pain	1.41 (1.04–1.90)	**<0.05**
Percentage of time spent on educational activities about the management of chronic pain	1.06 (1.04–1.06)	**<0.05**
Perception of the risks associated with short-acting opioids	1.15 (1.00–1.31)	0.05
Perception of the risks associated with long-acting opioids	0.88 (0.78–1.00)	0.06

**Notes**: Level of significance was set at 95%, with p-value ˂ 0.05 as statistically significant. Statistically significant p values are shown in bold. The dependent variable was comfort level, which was coded as a dichotomous variable (two groups: 0–6 and 7–10). ^a^Other settings included local community service centers, long-term care residences, outpatient clinics, rehabilitation centers, operating rooms, palliative care, and telemedicine. ^b^Remote resource regions as defined by Revenu Quebec (ie, the provincial revenue agency): Bas-Saint-Laurent, Saguenay-Lac-Saint-Jean, Abitibi-Témiscamingue, Côte-Nord, Nord-du-Québec, and Gaspésie-Îles-de-la-Madeleine. Non-remote regions are located near major urban centers. Variance inflation factor was ˂10 and tolerance was ˃ 0.1.

## Discussion

This study among a sample of 207 prescribers of the province of Quebec, Canada, was conducted to identify the factors that were associated with the comfort level of healthcare professionals in dispensing and adjusting prescriptions for the treatment of CP. To the best of our knowledge, this is the first study that compares the comfort level of physicians, pharmacists, and nurses when treating people with chronic non-cancer pain. More than half reported a comfort level in dispensing and/or adjusting prescriptions for the treatment of CP <6/10. The comfort level varied according to various socioeconomic and professional factors (profession, practice setting and region, years of clinical experience, continuing educational activities about the management of CP, living with CP, having a relative living with CP, and perception of opioid risk), which can lead to disparities in the quality of care and outcomes for patients.

Investigating the comfort level and sociodemographic and professional characteristics associated with the comfort level of healthcare professionals in prescribing medications for CP can unveil potential barriers that impact their confidence in managing individuals living with CP. For example, we found that even though most healthcare professionals (57%) had over 11 years of experience, less than 50% expressed a comfort level exceeding 6 when it came to prescribing and adjusting medications for the management of CP. Additionally, we found that healthcare professionals with over 11 years of experience exhibited a higher level of comfort than their counterparts who had practised for less than 11 years. This aligns with previous research, which demonstrated that healthcare professionals’ confidence in managing patients with CP is indeed influenced by their clinical experience.^24^ This phenomenon could be attributed to experiential learning and an enhanced understanding of pain management over time.^24^ It also points out an inadequate initial training of healthcare professionals regarding pain management. Literature indicates that the education of healthcare professionals concerning pain management is suboptimal, with pain-related topics often constituting a very small proportion of the medical curriculum.^25^,^26^ This study provides empirical evidence that the treatment of patients with CP is a substantial challenge for healthcare professionals. The identification of factors associated with comfort level is crucial and will help in developing focused training programs, ultimately enhancing the confidence of healthcare professionals in managing such cases.

Studying the characteristics of healthcare providers that are associated with their decisions regarding pain management is essential for understanding disparities in healthcare practices and implementing evidence-based and patient-centred care plans. One of the main reasons for the lack of confidence among healthcare professionals when dealing with CP is the knowledge gap and lack of training in this field, which has been identified as a barrier to effective pain management.^8^,^12–14^ Pain is a complex phenomenon that involves physiological and psychological components.^7^ Management of pain includes pharmacological, physical, and psychological treatments such as physiotherapy and cognitive behaviour therapy.^1^ Several studies have reported that involvement in continuing educational activities regarding pain management improves the clinical skills of care providers and ultimately better patient outcomes.^23^,^27–29^ We also found that the percentage of continuing educational activities about the management of pain is associated with the comfort level of healthcare professionals. Additionally, healthcare professionals working in remote regions reported lower levels of comfort than those working in non-remote regions. This could be due to fewer opportunities available for participation in continuing educational activities for healthcare professionals practising in remote regions of Quebec, Canada.^30^ Participating in continuing educational activities on pain, such as interdisciplinary approaches for the management of pain, non-pharmacological therapies for pain, exploring telemedicine, and devising a patient-centred care plan, not only enhance the confidence of healthcare professionals in treating CP patients but also contribute to better patient satisfaction.^18^,^27^ The efficacy of such educational activities has been demonstrated through initiatives like Project ECHO, a telemedicine-driven mentoring approach.^27^ Primary care physicians who participated in these sessions reported an increase in confidence when it came to managing patients with CP.^27^

In Canada and Quebec, physicians and nurse practitioners have the authority to prescribe various classes of medications for managing CP.^19^,^20^ Additionally, in Quebec, pharmacists can modify (adjust the dosage, form, or dose; extend an existing prescription) or discontinue pain medications.^20^ We found that nurse practitioners reported lower levels of comfort than physicians and pharmacists when prescribing or adjusting treatment for the management of CP. To the best of our knowledge, this is the first study that compares the comfort level of these three types of healthcare professionals. Effective pain management involves an interdisciplinary approach involving physicians and allied healthcare professionals. Nurse practitioners and pharmacists are the first point of contact for many people living with CP, particularly in regions with limited resources and when there is a long waiting time for appointments with physicians. Research has indicated that the median wait time for consultation with pain specialist is 6 months, but it can extend to as much as 3 to 5 years.^31–33^ Furthermore, it was shown that physicians do rely on nursing staff to assess and evaluate patients following the treatment of CP and report any adverse effects of pain medication.^25^ If these allied healthcare professionals are equipped with the skills and knowledge to confidently deal with patients living with CP, it will not only improve patient outcomes but will also reduce consultation and referral to physicians. We found that pharmacists were more comfortable than physicians in dealing with patients living with CP. Past research has shown that implementing a multidisciplinary strategy that includes the involvement of a pharmacist and nurses leads to improved patient outcomes in people with CP rather than relying solely on physicians.^34^,^35^ This study further emphasizes the importance of a multidisciplinary approach involving pharmacists and other allied health professionals when formulating treatment plans for patients treated for CP.

Analysis showed that sex at birth was associated with the comfort level of the prescribers (males reported higher levels of comfort than females). A few studies have reported that providers’ characteristics also influence their decisions about pain management,^18^,^24^ but this has not been explored in detail. Also, most have focused on physicians only and have not explored the characteristics and attributes of allied healthcare professionals that may influence their decision making. A recent study also showed that female physicians were less likely to prescribe opioids than their male counterparts.^36^ Certain inherent attributes, like those often associated with females, such as a tendency toward cautious decision-making, conservative treatment approaches and focus on goal prioritization, could also affect their comfort level when prescribing medication for people living with CP.^36^,^37^ Another example is a vignette study by Bartley EJ et al which showed that practitioner’s sex was related to pain management decisions and female practitioners were more likely to recommend pain treatment with non-opioid analgesics than males.^24^ Similarly, it has been shown that male and female healthcare providers tend to prescribe more analgesics to patients who have similar sex as the healthcare providers themselves.^9^,^38^ Our result is thus in accordance with literature.

### Strengths and Limitations

We were able to reach a diverse sample of healthcare professionals exhibiting variability in terms of their comfort levels in dispensing or adjusting prescriptions for CP treatment, participation in continuing education activities related to CP, years of practice, practice settings, and regions of residence. Despite this diversity, the majority of our participants identified as women (85.5%), which is a limitation of this study. When identifying characteristics associated with ≥6/10 comfort levels, the possibility of a type II error was minimized due to the substantial sample size (the 10 events per independent variable included in the logistic regression model rule was respected;^39^ 90 participants reported ≥6/10 comfort levels and no more than 9 factors were considered in the model). Additionally, it should be noted that the number of participants across the three healthcare professional categories (nurse practitioners, physicians, and pharmacists) was not evenly distributed in our sample, potentially affecting the representativeness of each profession in Quebec. External validity to healthcare professionals outside Quebec is also limited, as initial training, prescribing regulations, and guidelines can vary from one country to another. Furthermore, the comfort level is a subjective measurement that does not represent clinical competency. We did not consider factors linked to the work environment, such as practice guidelines. Further investigation on larger and more diverse datasets is needed to gain a comprehensive understanding of the characteristics associated with healthcare providers’ confidence that influence their decisions regarding the management of CP.

## Conclusion

In our study, less than half of healthcare professionals were comfortable with analgesic prescribing. The comfort level varied according to socioeconomic and professional factors, which can lead to disparities in the quality of care and outcomes for patients. Our results reinforce the importance of investing in initial training and continuing education of prescribers. Recognition of factors associated to the comfort levels of healthcare professionals could contribute to the development of targeted training programs. Further studies are needed to determine whether modifying pain education to target different prescriber characteristics would improve patient outcomes.

## Data Availability

The dataset is not publicly available because participants did not initially provide consent to open data but can be obtained from the corresponding author upon reasonable request and conditionally to a proper ethical approval for a secondary data analysis.
